# Understanding the Relation Between Early-Life Adversity and Depression Symptoms: The Moderating Role of Sex and an Interleukin-1β Gene Variant

**DOI:** 10.3389/fpsyt.2019.00151

**Published:** 2019-03-22

**Authors:** Robyn J. McQuaid, Robert L. Gabrys, Opal A. McInnis, Hymie Anisman, Kimberly Matheson

**Affiliations:** ^1^The Royal's Institute of Mental Health Research, University of Ottawa, Ottawa, Canada; ^2^Department of Neuroscience, Carleton University, Ottawa, ON, Canada

**Keywords:** cytokines, depression, inflammation, interleukin-1β, polymorphism, stress

## Abstract

Pro-inflammatory cytokines, such as interleukin (IL)-6 and tumor necrosis factor-α (TNF-α), are thought to play a fundamental role in the pathogenesis of depression within a subset of individuals. However, the involvement of IL-1β has not been as consistently linked to depression, possibly owing to difficulties in detecting this cytokine in blood samples or that changes in circulating levels might only be apparent in a subgroup of patients who have experienced early-life adversity. From this perspective, the association between early-life adversity and depressive illness might depend on genetic variants regulating IL-1β activity. Considering the inflammatory-depression link, and that women are twice as likely to experience depression compared to men, the current study (*N* = 475 university students) examined the moderating role of three independent cytokine single nucleotide polymorphisms (SNPs; IL-1β rs16944, IL-6 rs1800795 SNP, TNF-α rs1800629) in the relationship between early-life adversity and depressive symptoms, and whether these relations differed between males and females. The relation between childhood adversity and depressive symptoms was moderated by the IL-1β SNP, and further varied according to sex. Specifically, among females, higher childhood maltreatment was accompanied by elevated depressive symptoms irrespective of the IL-1β SNP, but among males, this relationship was particularly pronounced for those carrying the GG genotype of the IL-1β SNP. These findings suggest that, in the context of early life adversity, genetic variations of IL-1β functioning are related to depressive symptomatology and this may vary among males and females. The present study also, more broadly, highlights the importance of considering the confluence of experiential factors (e.g., early life adversity) and personal characteristics (e.g., sex and genetics) in understanding depressive disorders, an approach increasingly recognized in developing personalized treatment approaches to this illness.

## Introduction

The role of inflammation in the pathogenesis of depression is well-established (1, 2). Depressed individuals display elevated circulating pro-inflammatory cytokine levels (3) and administering pro-inflammatory cytokines to adults (e.g., during the course of treatment for hepatitis C and for some types of cancer) induces symptoms of depression, an effect that could be reversed by antidepressants (4). A meta-analysis comprising 58 studies indicated that interleukin (IL)-6 and the acute phase reactant C-reactive protein (CRP) were elevated in major depressive disorder, and to a lesser extent, tumor necrosis factor (TNF)-α (5). Treatment with antidepressants, as expected, was accompanied by reduced peripheral IL-6, TNF-α, and the anti-inflammatory IL-10 (6).

When considering the link between IL-1β and depression, the results have been mixed. Some meta-analyses do not find an overall association between IL-1β and major depression (3, 5, 7), although it was suggested that this could be due to measurement issues as concentrations of IL-1β are very low in blood (5). Moreover, no differences were found in basal IL-1β levels in a recent paper with MDD patients, but proteins upstream IL-1β production were elevated (8). However, others have reported elevated IL-1β levels in major depression (9), and a separate meta-analysis found elevated IL-1β in patients affected with major depressive disorder, although, this was only when the studies included were rated as having high quality methodologies (10). It was suggested that IL-1β changes may be apparent in a subgroup of patients, including individuals with a history of childhood trauma (10). Consistent with this perspective, early-life adversity might be centrally involved in the relationship between depression and inflammation by promoting changes to inflammatory signaling (11). In this regard, females who reported sexual abuse in adolescence displayed higher plasma levels of the pro-inflammatory cytokine IL-6 (12). Scores on an early trauma inventory scale were positively associated with serum IL-6, TNF-α and IL-1β levels (13), and depressed individuals with experiences of childhood trauma displayed higher peripheral cytokine levels compared to individuals with major depressive disorder who did not experience childhood trauma (14).

Beyond plasma cytokine levels, genetic variants of pro-inflammatory cytokines that can alter gene transcription and thereby affect inflammatory proteins, including IL-1β, have been associated with a wide range of physical and psychological disturbances, such as major depression (15, 16). Secretion of IL-1β protein can be influenced by a single nucleotide polymorphism (SNP), rs16944 located in the promoter region of the gene (17). However, currently there are inconsistent reports about whether the GG genotype (18) or the A/A genotype (19) is associated with elevated IL-1β levels. Moreover, other reports have suggested that the functional role of this SNP might depend on promoter region haplotypes (17, 20). Despite the poor understanding of the functionality of this SNP, it has been tied to a number of psychological outcomes. Specifically, individuals carrying two copies of the G allele, were found to have an early age of onset of depression (21), higher depressive symptoms and poorer response to antidepressant treatment (22–24), elevated cortisol levels following dexamethasone (25), and greater depressive symptoms following chronic interpersonal stressor encounters (26). However, there have also been some conflicting findings, such that an elevated risk of depression among the minor allele A carriers for individuals with schizophrenia spectrum disorders (27), Alzheimer's disease (28), and following childhood adversity (29).

Altered IL-6 expression has been linked to the IL-6 rs1800795 SNP, which is also located in the promoter region of the gene (30, 31). However, this SNP has been inconsistently linked to depression, in that individuals homozygous for the G allele exhibited an increase in depressive symptoms (32), whereas no association was found between this SNP and depression in other reports (33–35). It was found, however, that following interpersonal stress (26), and recent negative events (36), the G-carriers of this IL-6 SNP expressed fewer depressive symptoms relative to CC homozygotes. The TNF-α rs1800629 SNP located in the promoter region has also been inconsistently linked to depression and a recent meta-analysis found no significant association to depression (37). Only one previous investigation examined the TNF-α SNP in association to interpersonal stress and depression, and found no moderating role of the TNF-α SNP (26).

As women are approximately twice as likely to be diagnosed with depression (38), and report greater severity and increased symptoms compared to men (39), the pathophysiology of depression might also differ by sex. In fact, a large-scale gene expression study revealed multiple transcription changes in opposite directions between men and women with major depressive disorder (40).

Considering the discrepant findings regarding the IL-1β SNP, the primary aim of the current study was to examine the moderating role of the IL-1β SNP and that of IL-6, and TNF-α in the relation between early life adversity and depressive symptoms, and to determine whether any such effects would be moderated by sex. In the present investigation we chose to assess these factors in a sample of university students, as it has long been known that ~15–20% of students entering post-secondary educational institutions may have undiagnosed or subsyndromal symptoms of depression and anxiety (41). Moreover, early-life adverse experiences, such as childhood abuse and neglect, have been increasingly reported by college students and are thought to be contributing to large increases in the number of students presenting at university counseling services (42, 43). Given the considerable role for early life negative experiences in the evolution of adult depression, it was hypothesized that the GG carriers for the IL-1β SNP would display elevated depression scores following childhood maltreatment, although it was uncertain whether women or men might be more affected in this regard. Moreover, it was expected that the G-carriers of the IL-6 SNP would be linked to fewer depressive symptoms following childhood maltreatment compared to individuals with the CC genotype, but similarly, a sex effect was uncertain. The currently available data do not justify a hypothesis that early-life adversity and the TNF-α SNP would be associated with adult depression, but this SNP was included in the present analysis to replicate previous null findings.

## Methods

### Procedure

Students were recruited through the university's online computerized research system. Participants provided informed written consent, following which they completed measures of current depressive symptoms, experiences of early-life maltreatment, and a series of demographic questions (e.g., age, sex, and ethnicity). After completing the questionnaires, saliva samples were collected for later genotyping. Participants were debriefed and compensated with course credit. This study was approved by the Carleton University Ethics Committee for Psychological Research.

### Genotyping

Norgen collection kits (Norgen Biotek Corp., Thorold, Ontario Canada) were used for the collection of saliva samples for genotyping. Genomic DNA was extracted from the sample collection kit according to the manufacturer's instructions and diluted to approximately equal concentration (10 ng/μL). Genotyping was performed at the McGill University and Génome Québec Innovation Center (Montreal, Canada). Polymerase chain reaction (PCR) was used to amplify the DNA, and QIAXcel determined amplification status. To remove all unincorporated dNTPs, shrimp alkaline phosphatase was used. One probe per marker was used to do a single base extension and the product was desalted using 6 mg of resin. Products were spotted on a Sequenom 384-well chip using a Samsung Nanodispenser and then read by a Mass Spectrometer. A manual analysis was done for each marker. Primer sequences were: IL-1β forward: ACGTTGGATGCTGTCTGTATTGAGGGTGTG, IL-1β reverse: ACGTTGGATGATTTTCTCCTCAGAGGCTCC, IL-1β probe: GTGCTGTTCTCTGCCTC, TNF-α forward: ACGTTGGATGTTCTGGGCCACTGACTGATT, TNF-α reverse: ACGTTGGATGAAGGAAACAGACCACAGACC, TNF-α probe: AGGCTGAACCCCGTCC, IL-6 forward: ACGTTGGATGGATTGTGCAATGTGACGTCC, IL-6 reverse: ACGTTGGATGAGTGGTTCTGCTTCTTAGCG, and IL-6 probe: TGTGACGTCCTTTAGCAT.

### Measures

#### Depressive Symptoms

The 21-item Beck Depression inventory (BDI) (44) was used to assess depressive symptoms. For each item, participants selected one of four response options, which ranged from mild to severe indicators of depressive symptomatology. Total scores were calculated by summing across all items (α = 0.90).

#### General Health

Participants completed a one-item question asking, “*In your opinion, how do you describe your health*,” which ranged from one (poor) to five (excellent). This question was assessed to examine how BDI scores map onto other aspects of life/functionality. As expected, it was found that poorer health related to higher depressive symptoms, *r* = −0.476, *p* < 0.001.

#### Childhood Maltreatment

The 31-item Childhood Maltreatment Questionnaire (short form) (45) assessed levels of early life adversity, including psychological (α = 0.95), physical (α = 0.90), and sexual abuse (α = 0.97) as well as neglect (α = 0.85). Each item is rated from 1 (never) to 5 (very often) indicating the frequency of experiences. Total scores were calculated by obtaining the mean across all items (α = 0.95).

### Statistical Analyses

Statistical analyses were performed using SPSS for Windows 24.0 (SPSS Science, Chicago, Illinois, USA). Analyses assessing genotype differences on childhood maltreatment and depression scores were performed using a one-way analysis of variance (ANOVA), followed by Bonferroni corrected *t*-tests for any significant outcomes. Sex differences were examined using independent samples *t*-tests. Correlational analysis was performed using Pearson product moment correlations. To examine the moderating role of genotype and sex on the relationship between childhood maltreatment and depression scores, and any possible 3-way interactions, PROCESS (model 3) was used (46), in which moderations are tested in a single model. To be sure findings were not influenced by medications, such as anti-depressants, medications were included as a covariate in PROCESS and effects remained unchanged. Although it is ideal to keep all three genotypes separate whenever possible, as we have previously discussed in relation to OXTR genes [see (47) for a discussion on this topic], moderation analyses require a dichotomous moderator. For moderation analyses genotypes were collapsed into two groups to perform dominant tests because of power issues. A power calculation conducted using on online A-Prior sample size calculator for a Hierarchical Multiple Regression using three predictors in set A (Gender, Il-1B, and Childhood Maltreatment), one predictor in set B (3-way interaction term), a desired power level of 0.8, a *p*-value of 0.05 and the anticipated small effect size of 0.02. The power calculator provided a desired sample size of *N* = 388 (48). For the three main models conducted (one for each of the 3 SNPs), corrections for multiple testing were made such that models were considered significant if they were less than *p* = 0.0167, and effects remained significant.

## Results

### Participants and Descriptive Information

The original sample comprised 925 Carleton University first year students of various ethnic backgrounds. Population stratification effects were found, in which the IL-1β rs16944, TNF-α rs1800629, and IL-6 rs1800795 genotype distributions significantly differed according to ethnic groups, $\chi_{\left(16\right)}^{2}$ = 77.0, *p* < 0.001, $\chi_{\left(16\right)}^{2}$ = 39.1, *p* = 0.001, and $\chi_{\left(16\right)}^{2}$ = 209.5, *p* < 0.001, respectively. As an example of these differences, 97.3% of the Asian participants (Chinese, Japanese, Korean) displayed the GG genotype for the IL-6 SNP, whereas, only 36.3% of participants who reported European white ethnicity had this genotype. Moreover, 89.9% of participants who identified as black (African, Haitian, Jamaican, and Somali) displayed the GG genotype for IL-6 SNP, which was more in-line with Asian participants, whereas individuals who reported South Asian ethnicity (East Indian, Pakistani, Punjabi, and Sri Lankan) had 59.4% GG carriers. This example highlights the large discrepancies of genotype distributions across ethnic groups and the importance of conducting gene x environment analyses within a single homogeneous ethnic group. Accordingly, the current study only examined the largest homogeneous ethnic group, which comprised 475 individuals of European white ethnicity. Of these individuals, there were 343 females and 132 males with a mean age of 19.45 years (SE = 0.11, range = 17–35 years).

Among participants five individuals were taking anti-inflammatory medications (1.1%), 20 individuals were taking anti-depressant medication (4.2%), six individuals were taking anti-anxiety medications (1.3%), and 34.9% reported other medications, which largely included birth control, asthma and allergy medications.

### Genotype Distributions and Differences

Six individuals could not be genotyped for the IL-1β rs16944 SNP and the TNF- α rs1800629 SNP, whereas four individuals could not be genotyped for the IL-6 SNP, rs1800795. Genotype distributions and Hardy-Weinberg Equilibrium expectations are represented in Table 1.

**Table 1:** Genotype frequencies, distributions, and Hardy-Weinberg equilibrium expectations.

**Genotype distributions**	**GG**	**GA**	**AA**	**Hardy Weinberg equilibrium**
IL-1β (total)	197 (42%)	225 (48%)	47 (10%)	$\chi_{\left(1\right)}^{2}$ = 2.22, *p >* 0.05
Males	54 (41.2%)	61 (46.6%)	16 (12.2%)	$\chi_{\left(1\right)}^{2}$ = 0.04, *p >* 0.05
Females	143 (42.3%)	164 (48.5%)	31 (9.2%)	$\chi_{\left(1\right)}^{2}$ = 2.74, *p >* 0.05
	**GG**	**GA**	**AA**	
TNF-α (total)	324 (69.1%)	133 (28.4%)	12 (2.6%)	$\chi_{\left(1\right)}^{2}$ = 0.1, *p >* 0.05
Males	93 (71.0%)	34 (26.0%)	4 (3.1%)	$\chi_{\left(1\right)}^{2}$ = 0.17, *p >* 0.05
Females	231 (68.1%)	99 (29.3%)	8 (2.4%)	$\chi_{\left(1\right)}^{2}$ = 0.47, *p >* 0.05
	**GG**	**GC**	**CC**	
IL-6 (total)	171 (36.3%)	225 (47.8%)	75 (15.9%)	$\chi_{\left(1\right)}^{2}$ = 0, *p >* 0.05
Males	39 (38.7%)	67 (46.3%)	24 (15.0%)	$\chi_{\left(1\right)}^{2}$ = 0.26, *p >* 0.05
Females	132 (30.0%)	158 (51.5%)	51 (18.5%)	$\chi_{\left(1\right)}^{2}$ = 0.11, *p >* 0.05

There were no differences in childhood maltreatment total scores across genotypes for the IL-1β, *F*_(2, 466)_ = 0.69, *p* = 0.50, TNF-α, *F*_(2, 466)_ = 0.14, *p* = 0.87, or IL-6 SNPs, *F*_(2, 468)_ = 0.41, *p* = 0.67. Depression scores, however, varied with the IL-1β genotype, *F*_(2, 466)_ = 4.00, *p* = 0.02, η^2^ = *0.0*2, with GG carriers reporting more severe depressive symptoms (*M* = 10.23, *SE* = 0.57), compared to GA carriers (*M* = 8.11, *SE* = 0.54), *p* = 0.02, whereas AA carriers did not significantly differ compared to the other groups. Depression scores, in contrast, did not vary based on the TNF-α genotypes, *F*_(2, 466)_ = 0.28, *p* = 0.76, or the IL-6 SNP, *F*_(2, 468)_ = 0.84, *p* = 0.43. Table 2 shows means for genotype groups collapsed according to the dominant model.

**Table 2:** Mean and standard deviation of childhood maltreatment and depressive symptoms collapsed across genotype groups.

**Genotype groups**	**Childhood maltreatment (*M ± SD*)**	**Depressive symptoms *(M ± SD)***
**IL-1β**		
Males (GG)	1.32 (±0.34)	6.78 (±7.66)
Males (GA/AA)	1.48 (±0.46)	7.74 (±7.37)
Females (GG)	1.59 (±0.63)	11.53 (±8.96)
Females (GA/AA)	1.49 (±0.56)	8.74 (±7.37)
**TNF-α**		
Males (GG)	1.46 (±0.46)	8.04 (±8.00)
Males (GA/AA)	1.30 (±0.27)	5.63 (±5.75)
Females (GG)	1.51 (±0.59)	9.50 (±8.14)
Females (GA/AA)	1.57 (±0.60)	10.78 (±8.30)
**IL-6**		
Males (GG)	1.42 (±0.33)	7.59 (±6.94)
Males (GC/CC)	1.40 (±0.45)	7.00 (±7.42)
Females (GG)	1.50 (±0.05)	10.32 (±0.74)
Females (GC/CC)	1.55 (±0.58)	9.65 (±8.00)

### Correlations

As expected, females report more severe depressive symptoms than males, *t*_(1, 473)_ = 3.19, *p* = 0.002 (females *M* = 9.92, *SE* 0.44; males *M* = 7.32, *SE* 0.65), and also reported higher levels of childhood maltreatment, *t*_(1, 332.0)_ = 2.52, *p* = 0.012 (females: *M* = 1.53, *SE* 0.03; males: *M* = 1.41, *SE* 0.04). As shown in Table 3, the relations between the childhood maltreatment total scores and depressive symptoms were significant, as were the relations between the different forms of maltreatment and depressive scores (reports of sexual abuse were very infrequent and as such, this subscale was not assessed as an independent measure).

**Table 3:** Pearson correlations among childhood maltreatment total and subscales and depressive symptoms.

	**1**	**2**	**3**	**4**	**5**
1. Depressive symptoms	––––––			
2. Childhood maltreatment (total score)	0.47^**^	––––––			
3. Childhood physical abuse	0.31^**^	0.78^**^	––––––		
4. Childhood psychological abuse	0.48^**^	0.96^**^	0.63^**^	––––––	
5. Childhood neglect	0.41^**^	0.87^**^	0.65^**^	0.77^**^	––––––

** *p < 0.001*.

### Moderation Analyses

**Figure 1:**
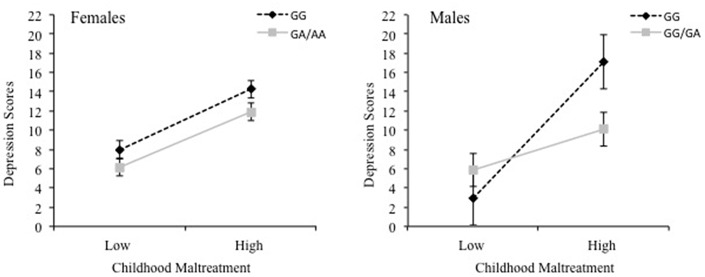
The 3-way interaction between childhood maltreatment, the IL-1β rs16944 SNP (GG vs. GA/AA) and sex in relation to depression scores. Females are shown in the left graph and males are shown in the right graph.

To examine whether sex and cytokine SNPs moderated the relationship between childhood maltreatment and depression scores, moderation analyses were conducted separately for each cytokine SNP. For IL-1β, the overall model was significant, *R*^2^ = 0.26, *F*_(7, 461)_ = 23.11, *p* < 0.001, revealing interactions between Childhood Maltreatment × IL-1β, *p* = 0.03, Childhood Maltreatment × Sex, *p* = 0.005, and IL-1β × Sex, *p* = 0.003, as well as a significant 3-way interaction between Childhood maltreatment × IL-1β × Sex, $R_{change}^{2}$ = 0.01, *F*_(1, 461)_ = 7.86, *p* = 0.005. The findings of the 3-way interaction are shown in Figure 1, wherein a positive relationship between childhood maltreatment and depressive symptoms for females was significant for both GG and A carriers of the IL-1β SNP, *B* = 6.78, *t* = 7.27, *p* < 0.001, 95% CI [4.95, 8.61] and *B* = 6.16, *t* = 6.84, *p* < 0.001, 95% CI [4.39, 7.93], respectively. For males, the relationship between childhood maltreatment and depression was also significant for both genotype carriers, however, it was a much stronger effect for GG carriers, *B* = 15.17, *t* = 5.36, *p* < 0.001, 95% CI [9.61, 20.73], compared to A carriers *B* = 4.55, *t* = 2.60, *p* = 0.01, 95% CI [1.11, 7.98]. In fact, among males with low levels of childhood maltreatment, depression scores were low irrespective of the genotype. However, as levels of maltreatment increased, depression scores were appreciably higher, and more so among those with the GG alleles than among A carriers.

Due to the significant IL-1β SNP model when examining total child maltreatment scores, we conducted follow-up analyses for the different forms of abuse. Once more, 3-way interactions between IL-1β x Sex and each of the subscales of childhood maltreatment were evident: psychological maltreatment, $R_{change}^{2}$ = 0.01, *F*_(1, 461)_ = 5.43, *p* = 0.02, neglect, $R_{change}^{2}$ = 0.01, *F*_(1, 461)_ = 5.57, *p* = 0.02, and physical abuse, $R_{change}^{2}$ = 0.01, *F*_(1, 461)_ = 4.34, *p* = 0.04. However, after correcting for multiple testing, these effects were no longer considered significant at *p* < 0.0167.

Analysis of the moderating relationships involving the TNF-α and IL-6 SNPs indicated that none of these interactions were significant.

## Discussion

The current study revealed that, although the effect size was small, depressive symptoms were higher among individuals with the GG genotype of the IL-1β rs16944 SNP, an outcome that was most pronounced among those who experienced childhood adversity. These findings align with earlier reports that the GG carriers show an earlier age of onset of depression (21), enhanced severity of depressive symptoms (24) and poorer responses to antidepressants (22–24). Moreover, individuals with the GG genotype also displayed greater depressive symptoms following chronic interpersonal stressor experiences (26). Thus, the current findings replicate this earlier report showing that the relation between childhood maltreatment and depressive symptoms was also stronger among individual homozygous for the GG genotype. However, it has also been reported that individuals with the GG genotype displayed lower depression scores following childhood adversity (29). Why these differences exist are uncertain, however, in Kovacs et al. report the relations to depression were only apparent among individuals who encountered the most marked childhood adversity. Maltreatment scores in the current study were modest and thus, comparisons or generalizations beyond this population would be inappropriate.

As commonly observed, depressive symptoms were higher in women than in men. Sex also interacted with the IL-1β SNP and childhood maltreatment in predicting depressive scores. Specifically, following maltreatment, males who carried the GG genotype of the IL-1β SNP showed particularly marked depressive symptoms. This finding appears to be in line with research examining systemic inflammation and sex, wherein a higher production of pro-inflammatory cytokines were associated with increasing depressive symptoms for males, however females exhibited reduced pro-inflammatory cytokines as depressive symptoms increased (49). It appears that there are gender differences in the relation between depressive symptoms and inflammatory patterns. Additionally, there have been several reports indicating that the sex differences in depression varied with SNPs on genes other than those coding for cytokines. For instance, the glucocorticoid receptor gene (NR3C1) SNP rs6195 was related to depression among females, but not males (50). Moreover, there was a sex-dependent moderation of a functional mineralocorticoid receptor (MA) haplotype in the relationship between childhood maltreatment and depression, revealing that males in the clinical sample were at increased risk of depression (51). As well, among boys and girls that carried the short allele of the serotonin transporter gene, 5-HTTLPR, displayed opposite responses to environmental stressors (52). In line with these reports, a meta-analysis suggested that pronounced gene expression differences exist between men and women with major depression, and even more relevant to the current findings is the suggestion that treatments aimed at suppressing immune function might be more appropriate for men with depression compared to women (40).

These findings highlight the importance of considering individual characteristics, such as sex, when examining the link between genetic variants and depression, and the treatment of this disorder. Personalized treatment approaches extend beyond this to also recognize the importance of contextual and environmental factors, such as early-life stressful experiences (53). Early-life stressors and/or trauma have been strongly linked to the later development of depression and may interact with individual genetic variants to contribute to depression (54). By example, it was reported that interactions between early-life adversity and the oxytocin receptor gene (OXTR) SNP rs5657 (55), and brain derived neurotrophic factor (BDNF) SNP (56, 57) were linked to depressed mood. Interactions have also been reported between early-life maltreatment and a polymorphism in the promoter region of the serotonin transporter gene (5-HTTLPR) (58). To be sure, considerable controversy exists concerning the reliability of such reports (59), but it should be considered that there are differences in sample characteristics and contexts, such as the timing and type of stress experienced across studies, which highlight the importance of accurate characterization of the environmental exposures underlying the gene × environment effects (54, 60).

In the current study general depressive symptoms were examined, but it would be of particular interest to focus on specific symptoms or subtypes of depression, such as atypical depression, which might be differentially related to sex and/or the cytokine SNPs. As well, specific cytokine SNPs that were not tied to depression might be more strongly linked to suicide behaviors. In this regard, the GG genotype of the TNF-α SNP, rs1800629, was associated with increased risk for suicide attempts among patients with major depressive disorder (61), even though this TNF-α SNP was not linked to general depression and depressive symptomatology (26, 37). The current findings similarly did not show an association between this TNF-α SNP and depressive scores.

Although there have been several reports indicating that inflammatory factors, notably circulating IL-6 and TNF-α, were associated with major depressive illness (5, 62), as previously observed (33–35), the IL-6 rs1800795 SNP was not directly related to depression scores in the present study. Moreover, the IL-6 SNP did not interact with childhood maltreatment or sex to predict depression symptoms. This differs from earlier reports showing interactions between this SNP with interpersonal stress (26), and recent negative events (36), in relation to depression. Of course, early-life maltreatment ought to be distinguished from recent and/or acute stressors, which could explain the non-significant effects in the current study. Recently, this IL-6 SNP has been found to influence antidepressant treatment outcomes in major depressive patients, which might suggest a role for IL-6 in treatment resistant depression (63). As described earlier, support for IL-6 involvement in depression has also come from the many reports showing elevated levels of peripheral IL-6 in depressed patients (5). However, plasma cytokine levels do not necessary reflect levels of this cytokine within the brain. There is, indeed, reason to believe that inflammatory factors released from microglia (64, 65) may contribute to psychiatric disorders, including depression (66). However, it is unlikely that these cytokine variations can be detected in plasma, or that circulating cytokine levels would parallel brain cytokine variations [e.g., (67)].

There are several limitations associated with the current study. Specifically, early-life maltreatment was based on retrospective self-reports, and although this is common when examining childhood adversity, reports might be biased by the individuals' current affective state, and individuals could be unaware of events that took place years earlier. The current study also examined depressive symptoms among university students and thus the findings cannot be generalized to a clinically depressed population. Moreover, it should be noted that the mean scores for both childhood maltreatment and depressive symptoms were fairly low and the findings could differ within the context of higher maltreatment and depressive scores. An additional limitation concerned the relatively small sample size, even though it was determined that the sample size for the current study (*N* = 495 individuals of a homogeneous ethnicity) provided sufficient power to detect small effects, and significant effects survived correction for multiple testing. Due to population stratification effects, we had to limit our analyses to the largest homogenous ethnic group, which comprised individuals who identified as European/white. A larger sample ideally might have allowed for assessment of gene x environment variations among other ethnic groups. This is particularly of interest when we consider the importance of culture in personalized medicine (68, 69). Moreover, there is a possibility that the different cytokine SNPs assessed independently might be additively or interactively linked to depressive symptoms. For instance it has been reported that BDNF and 5-HTTLPR interactions were additive in predicting lifetime depression diagnosis (70), however, in the current sample we had limited power to add additional factors to our model. It is also problematic that the functionality of the IL-1β SNP is not well-understood (25). It would have been ideal to measure the relation between the rs16944 SNP frequency and gene expression levels (i.e., the expression quantitative trait loci [eQTLs; (71, 72)]. This approach could potentially facilitate a better understanding of the functional role of the IL-1β SNP. However, human eQTL studies typically involve analyses in blood-derived cells, as opposed to the saliva samples that were used in the present study, and it also appears that gene expression might vary with different cell types (71). Despite the potential benefits of eQTL analyses, the present data do not lend themselves to this approach. Finally, the current study precludes any causal effects to be inferred.

Together with earlier findings, the present report indicates that the IL-1β rs16944 SNP was associated with depressive symptoms. The current report shows that this relationship might be dependent on environmental contexts, including early-life maltreatment and individual characteristics, such as sex. In fact, sex differences appear to be especially critical in the relation between SNPs and depression, as new reports highlight gene-expression changes in opposite directions for men and women with major depression. These findings, together with other reports, highlight the importance of the social determinants of health when examining depression, an approach increasingly recognized in personalized approaches to medicine.

## Author Contributions

RM, OM, and HA contributed to the conception and design of the current experiment. Testing and data collection were performed by RM and OM. Data analysis and the writing of the manuscript were performed by RM, RG, HA, and KM. All authors approved the final version of the paper for submission.

### Conflict of Interest Statement

The authors declare that the research was conducted in the absence of any commercial or financial relationships that could be construed as a potential conflict of interest.
